# Exploiting urine-derived induced pluripotent stem cells for advancing precision medicine in cell therapy, disease modeling, and drug testing

**DOI:** 10.1186/s12929-024-01035-4

**Published:** 2024-05-09

**Authors:** Xiya Yin, Qingfeng Li, Yan Shu, Hongbing Wang, Biju Thomas, Joshua T. Maxwell, Yuanyuan Zhang

**Affiliations:** 1 Department of Plastic and Reconstructive Surgery, Shanghai Ninth People’s Hospital, Shanghai Jiao Tong University School of Medicine, Shanghai, China; 2 Department of Burn and Plastic Surgery, West China Hospital, Sichuan University, Chengdu, China; 3 Department of Pharmaceutical Sciences, School of Pharmacy, University of Maryland, Baltimore, Baltimore, MD USA; 4 Keck School of Medicine, Roski Eye Institute, University of Southern California, Los Angeles, CA 90033 USA; 5 Wake Forest Institute for Regenerative Medicine, Wake Forest University Health Sciences, Winston-Salem, NC USA

**Keywords:** Urine-derived stem cell, iPSCs, Disease modelling, Cell therapy, Drug testing, Regenerative medicine, Personalized medicine

## Abstract

The field of regenerative medicine has witnessed remarkable advancements with the emergence of induced pluripotent stem cells (iPSCs) derived from a variety of sources. Among these, urine-derived induced pluripotent stem cells (u-iPSCs) have garnered substantial attention due to their non-invasive and patient-friendly acquisition method. This review manuscript delves into the potential and application of u-iPSCs in advancing precision medicine, particularly in the realms of drug testing, disease modeling, and cell therapy. U-iPSCs are generated through the reprogramming of somatic cells found in urine samples, offering a unique and renewable source of patient-specific pluripotent cells. Their utility in drug testing has revolutionized the pharmaceutical industry by providing personalized platforms for drug screening, toxicity assessment, and efficacy evaluation. The availability of u-iPSCs with diverse genetic backgrounds facilitates the development of tailored therapeutic approaches, minimizing adverse effects and optimizing treatment outcomes. Furthermore, u-iPSCs have demonstrated remarkable efficacy in disease modeling, allowing researchers to recapitulate patient-specific pathologies in vitro. This not only enhances our understanding of disease mechanisms but also serves as a valuable tool for drug discovery and development. In addition, u-iPSC-based disease models offer a platform for studying rare and genetically complex diseases, often underserved by traditional research methods. The versatility of u-iPSCs extends to cell therapy applications, where they hold immense promise for regenerative medicine. Their potential to differentiate into various cell types, including neurons, cardiomyocytes, and hepatocytes, enables the development of patient-specific cell replacement therapies. This personalized approach can revolutionize the treatment of degenerative diseases, organ failure, and tissue damage by minimizing immune rejection and optimizing therapeutic outcomes. However, several challenges and considerations, such as standardization of reprogramming protocols, genomic stability, and scalability, must be addressed to fully exploit u-iPSCs’ potential in precision medicine. In conclusion, this review underscores the transformative impact of u-iPSCs on advancing precision medicine and highlights the future prospects and challenges in harnessing this innovative technology for improved healthcare outcomes.

## Introduction

Initially, stem cell research was primarily centered on harnessing the potential of ESCs, which were derived from human embryos. Despite the tremendous promise of ESCs, their use became mired in ethical and political controversy due to the necessity of embryo destruction for their procurement [1, 2]. However, in 2006, a momentous breakthrough emerged from Kyoto University in Japan, led by Shinya Yamanaka and his team. They achieved a transformative feat by successfully reprogramming adult mouse fibroblast cells into a pluripotent state by introducing a set of four specific transcription factors: Oct4, Sox2, Klf4, and c-Myc. These reprogrammed cells earned the name ‘induced pluripotent stem cells’ (iPSCs) [3]. Building on this achievement, Yamanaka’s team extended their reprogramming method to human cells in 2007, using the same quartet of transcription factors to create human iPSCs [4]. Induced pluripotent stem cells (iPSCs) are pluripotent stem cells generated from patients’ own somatic cells by reprogramming them to an embryonic stem cell-like state, making it possible to create a patient-specific disease model customized for their genetic information without raising similar ethical considerations of embryonic stem cells (ESCs) [5]. iPSCs have the potential to differentiate into three germ layers and generate a broad spectrum of cell types in the body, suitable as an appreciable tool for regenerative medicine. Meanwhile, the patient-specific origin of iPSCs leads to the eliminated risk of immune rejection and enhanced effectiveness while used in autologous cell therapy. iPSCs can be generated from diverse readily procurable cell sources like dermal fibroblasts and peripheral blood mononuclear cells (PBMCs) from an extensive variety of populations, expanding the scope of research models [6, 7]. iPSCs show unique values in investigating rare and genetic diseases when relevant animal models are scarce, as the introduction of specific genetic mutations assists in studying the progression of diseases related to individuals [8]. In brief, iPSCs demonstrate massive benefits in regenerative medicine, drug discovery, and disease modeling.

Despite the merits, there are several challenges in the research of iPSCs. The efficient induction of iPSCs requires rigorously regularized protocols, which has not reached a common sense among diverse laboratories. The incomplete differentiation of iPSCs may cause the formation of teratomas, provoking uncertainties regarding the safety issue in the utilization of iPSCs. Additionally, the generation of iPSCs touches on ethical and regulatory apprehensions concerning the invasive acquisition procedure of cell sources from patients [8]. From the aspect of clinical transition, the determination of an easily obtainable cell source for large-scale production, and ways to minimize the cost for iPSC generation and differentiation remain unsettled troubles.

A proper cell source is vital for the generation and application of iPSC. The cell source should be easily acquirable from consistent donors, which is more reproducible and reliable with fewer risks of variability. Regarding cell features, the cell source with high reprogram ability and proliferative capacity is more efficient for the generation of iPSC. To ensure its safety in patients, the cell source should possess genetic and epigenetic stability, with no tendency of tumorigenicity. In addition, the tissue origin is pivotal due to the related obtaining process, ethical concerns, and compatibility with research goals.

Urine-derived stem cells (USCs) are a type of adult stem cell derived from body fluids that can be isolated from urine samples, a waste product that is routinely discarded [9]. Unlike skin or blood cells, the non-invasive collection of USCs eliminates the need for invasive surgical procedures, reducing patient discomfort and potential risks associated with tissue harvesting. USCs have demonstrated excellent proliferative capacity, self-renewal ability, immunomodulatory properties [10, 11], and the ability to differentiate into various cell types, including but not limited to neurons, bone cells, muscle cells, and cartilage cells [12, 13]. These unique characteristics make USCs particularly advantageous as a cell source for iPSCs.

Urine-derived iPSCs (u-iPSCs) are a subtype of iPSCs generated from cells in urine samples [14], mainly from USCs [15, 16]. As a cell source of patient-specific pluripotent cells, u-iPSCs can be obtained at low cost with a non-invasive and patient-friendly method and offer several advantages over other sources of iPSCs. USCs can be reprogrammed into iPSCs and then differentiated into various cell types. USCs reprogram into iPSCs more efficiently and rapidly than other somatic cells. They achieve of 80% transduction rate compared to 50% in mesenchymal cell lines. USC-derived iPSCs show morphological changes indicative of reprogramming within 3 days, form distinct colonies expressing pluripotency markers by day 7, and reach maturity by day 10–14, whereas mesenchymal cell-derived colonies require 28 days [17]. The shorter induction time and higher reprogramming efficiency owes to epithelial origin of USCs, which means the elimination of mesenchymal-to-epithelial transition (MET) process [12, 16, 17]. U-iPSCs have been adopted in the research of precision medicine, especially the establishment of patient-specific disease modelling, including neuromuscular, neurodegenerative, cardiovascular, hematopoietic, and pediatric diseases.

## Advancing technology for the generation of u-iPSCs

**Fig. 1:**
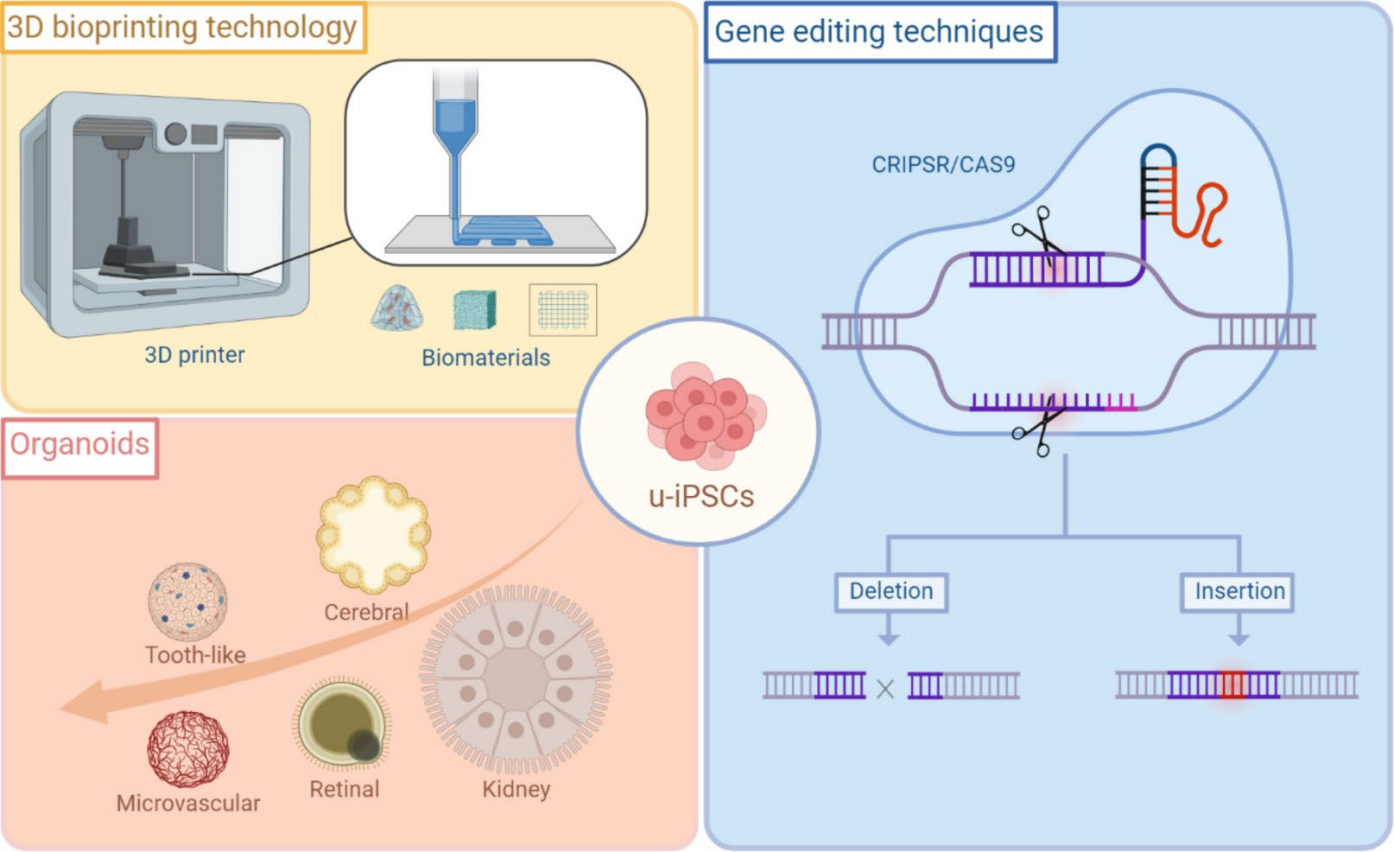
Advancing techniques in utilizing u-iPSCs. 3D printing techniques, organoids and gene editing techniques are three emerging technologies in the study of u-iPSCs. iPSCs can be used in 3D bioprinting to create patient-specific organoids for transplantation, which could address the shortage of organ donors and reduce the risk of transplant rejection. Moreover, gene editing techniques like CRISPR/Cas9 can be used to correct genetic mutations in iPSCs before they are used in therapeutic applications. (Created with BioRender.com)

Over the subsequent years, the realm of iPSC research witnessed rapid expansion and diversification (Fig. 1). Researchers devised a variety of techniques for generating iPSCs, incorporating alternative transcription factors and non-viral delivery approaches. These innovations significantly improved the efficiency and safety of iPSCs production [18].

### Gene-editing techniques

Despite the reprogramming methods mentioned above, new technologies have been applied in the research of u-iPSC, such as using genome editing to directly correct genetic mutations and develop novel gene therapy methods. Gene editing technologies, such as CRISPR/Cas9 system [3, 4, 19], zinc-finger nuclease (ZFN) [18, 20, 21], and transcription activator-like effector nucleases (TALENs) [22], allow the direct insertion, deletion, or replacement of distinct DNA sequences, creating specific modification in patients’ genome at targeted locations. Recent studies have focused on generating gene-edited urine-derived induced pluripotent stem cells (u-iPSCs) from patients with various diseases, aiming to explore novel gene therapy approaches. Zou et al. successfully generated u-iPSCs from the urine of an achondroplasia (ACH) patient and corrected the Gly380Arg mutation using CRISPR-Cas9, thereby restoring the chondrogenic differentiation ability of ACH iPSCs [23]. Similarly, Zhou et al. generated u-iPSCs from spinal muscular atrophy (SMA) patients and converted the survival motor neuron 2 (SMN2) gene to a survival motor neuron 1 (SMN1)-like gene using CRISPR/Cpf1 and single-stranded oligodeoxynucleotides (ssODN). The resulting motor neurons (iMNs) from modified u-iPSCs exhibited rescued expression of SMN proteins [24]. In addition, Neumeyer et al. used the piggyBac DNA transposon system to integrate the human F8 gene into the genome of u-iPSCs derived from individuals with hemophilia A. Upon differentiation of the modified u-iPSCs into endothelial cells, they formed vascular networks and demonstrated the capacity to produce functional FVIII when implanted into the subcutaneous tissue of hemophilic mice [25]. Furthermore, Zeng et al. reprogrammed urinary stem cells (USCs) collected from Duchenne muscular dystrophy (DMD) patients with an exon 50 deletion into u-iPSCs [26]. Subsequently, they used TALEN-based nickases to integrate a functional mini-dystrophin gene into the rDNA locus of the u-iPSCs. Mini-dystrophin expression was detected both in the genetically modified u-iPSCs and in the cardiomyocytes differentiated from them.

It’s worth noting that due to the non-specific action of gene editing tools, they may inadvertently modify DNA regions that are like, but not the intended target gene. This can lead to the generation of unknown genetic variations. While researchers can use DNA sequencing to determine whether changes have occurred in off-target regions, there remains a certain level of risk to patient safety in clinical applications. To better protect patients, the precision and specificity of gene editing tools have been continuously improved and refined [4, 27]. The use of gene editing technology is also subject to strict legal regulations and ethical considerations.

### Organoids

Dissimilar to traditional two-dimensional (2D) in vitro cell culture which is incapable of mimicking the natural environment in vivo, an organoid is sophisticated designed using various cells, possessing the three-dimensional (3D) structure of a tissue or organ, reflecting the complex cell-cell communications and tissue interactions, and resembling in vivo functions [28]. iPSC-based organoids can be generated with cells obtained from specific patients, which provides a disease-targeted model for researchers to conduct a multitude of experiments on the underlying mechanisms of specific disease.

U-iPSC [29], as a non-invasive, readily available, consistent cell source with high proliferation capacity, reprogramming efficiency, low risks, and no ethical consequences, have been applied in the field of organoid research. Mulder et al. induced u-iPSCs from infant and pediatric urine with episomal vectors and generated human kidney organoids after rigorously characterization of their pluripotency and karyotyping [30]. Kim et al. investigated the effect of Matrigel and Y-27,632 on promoting self-renewal and differentiation capacity of USCs and successfully generated kidney organoid and hematopoietic progenitor cells from u-iPSCs [31]. To investigate the pathophysiological mechanisms of glomerular diseases, a u-iPSC based kidney organoid was developed by Nguyen et al. with artificially induced injuries using puromycin aminonucleosides (PAN) [32]. An interconnected network related to inflammation and cell death was confirmed, revealing the potential of u-iPSC based kidney organoid in regenerative medicine for kidney diseases.

Despite kidney organoids, u-iPSC have been utilized in the development of retinal organoids [33], microvascular grafts [25], cerebral organoids [34] and tooth-like structures [35]. Li et al. formed 3D retinal organoids with properly layered neural retina containing all retinal cell types by differentiating u-iPSCs into retinal fates. Notably, u-iPSCs produced highly mature photoreceptors, including red/green cone-rich photoreceptors, without the supplementation of retinoic acid [33].

Neumeyer et al. genetically modified u-iPSCs with full-length F8 and differentiated them into endothelial cells (ECs). These cells produced high levels of FVIII and self-assembled into vascular networks upon subcutaneous implantation into hemophilic mice, effectively correcting the clotting deficiency and offering a potential autologous ex vivo gene-therapy strategy for HA treatment [25]. Teles et al. generated three-dimensional human cerebral organoids with neurons and astrocytes differentiated from u-iPSCs derived from Down syndrome (DS) patients [34], demonstrating the developmental dynamics of the early-stage forebrain. In the study by Cai et al., u-iPSCs were differentiated into epithelial sheets and combined with mouse dental mesenchymes, resulting in tooth-like structures within 3 weeks with a success rate of up to 30% across 8 iPSC lines, comparable to hESCs. These structures contained enamel-secreting ameloblasts with physical properties resembling human teeth [35].

## 3D bioprinting technology

The combination of stem cells with other emerging technologies, such as 3D bioprinting and nanotechnology, has been adopted to create novel regenerative medicine strategies. iPSCs, derived from a patient’s own dermal fibroblasts or peripheral blood mononuclear cells, offer a sustainable source of cells for 3D printing. Scientists have achieved success in utilizing bio-inks containing human iPSCs to 3D print a wide array of tissues and organs, including but not limited to cartilage [36], skin [37], heart, liver [38], and neural tissues [39]. These tailored biological constructs not only cater to individual patient needs but also account for their unique genetic variations, thereby markedly reducing the risk of rejection. They hold substantial promise for playing a more prominent role in tissue repair and regeneration. In the realm of 3D printing technology, it can create intricate structures of tissues and organ models, faithfully replicating the microenvironments found in actual human diseases. Consequently, this technology is instrumental in disease modeling. In the domain of cardiovascular diseases, iPSC-derived cardiac cells have proven to be effective in emulating conditions such as dilated cardiomyopathy and myocardial infarction [40]. Within the context of neurodegenerative diseases, the utilization of 3D bioprinting with iPSCs has given rise to disease models for conditions such as Alzheimer’s [41], Parkinson’s [42], and amyotrophic lateral sclerosis (ALS).

These models serve to explore the interactions among various types of nerve cells, decipher the pathophysiological characteristics of diseases, and delve into the mechanisms underlying disease onset. For oncological diseases, 3D bioprinting with cell lines derived from iPSCs can construct structures resembling tumors, such as spheroids or organoids, faithfully simulating the tumor microenvironment. These models serve as a platform for investigating the different stages of cancer progression following transplantation into animal models [43]. Moreover, artificial skin models created through 3D bioprinting, using iPSCs as a foundation, maintain intricate cellular pathways, interactions between cells, and the interplay between cells and their microenvironment [44]. This attribute confers substantial research value, especially in the realms of drug toxicity testing and the evaluation of cosmetic products.

Using 3D-printing technology, USCs have been combined with various biomaterials as a construction and applied to the research of bone tissue regeneration and repair. While 3D-printing technology allows for personalized bone substitutes, it lacks the ability to regulate the topological morphology of the scaffold surface, which is crucial for stem cell behavior. The fabricated poly(e-caprolactone) (PCL) scaffold with nanoridge patterns constructed by Xing et al. enhanced protein adsorption and mineralization compared to bare PCL scaffolds. Loaded with USCs, these scaffolds showed increased proliferation, cell length, and osteogenic gene expression, indicating improved bone regeneration capability [45]. Zhang et al. built a 3D-printed polylactic acid and hydroxyapatite (PLA/HA) composite scaffold loaded with USCs in treating skull defects in a rat model. Evaluation at 4, 8, and 12 weeks revealed that the PLA/HA scaffold with USCs significantly promoted new bone regeneration, with nearly complete coverage of the defect area observed at 12 weeks. These results underscore the potential of 3D-printed scaffolds with USCs in bone tissue engineering [46]. For now, studies considering u-iPSCs as a cell source for 3D printing remain scarce. Shao et al. successfully generated u-iPSCs and differentiated them into neural stem cells (NSCs). The 3D printed scaffold loaded with these NSCs showed preferable efficacy in repairing spinal cord injury after transplanted into mouse models, indicating the potential of u-iPSC in tissue regeneration and repair [47]. We look forward to more related research to further confirm the application value of u-iPSCs.

## Applications of u-iPSCs in precision medicine

**Fig. 2:**
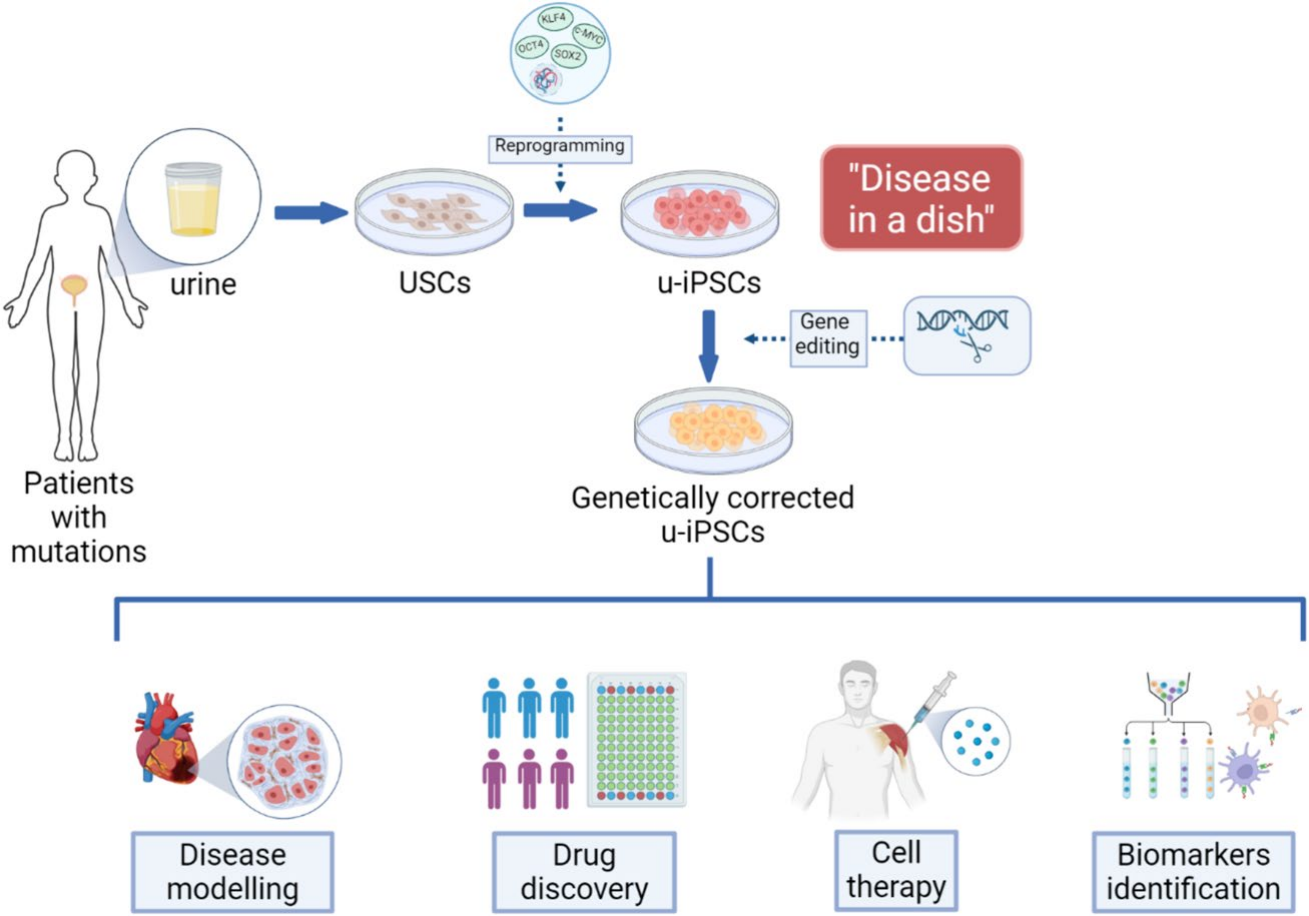
Disease modelling using u-iPSCs. USCs are harvested from urine of patients with specific mutations, and reprogrammed into u-iPSCs, which reflects the pathological condition under laboratory settings. Gene editing tools, such as CRISPR/Cas9, can be used to correct the genetic mutation in the patient’s u-iPSCs. The modified u-iPSCs are subsequently used in disease modelling, drug discovery, cell therapy and biomarker identification. (Created with BioRender.com)

U-iPSCs have the potential to revolutionize precision medicine by enabling personalized approaches to cell therapy, drug testing, and disease modeling **(**Fig. 2).

### Cell therapy

In the field of regenerative medicine, stem cells are widely used for tissue repair, regeneration, and cell therapy for various diseases due to their ability to self-renew and differentiate into various specific cell types [48]. Our team have previously published a series of studies where the efficacy and mechanism of USC-based cell therapy in various diseases such as Type 2 diabetic erectile dysfunction and complications [49–52], bladder diseases [53, 54], acute and chronic kidney injuries [55, 56], male infertility [2, 57], and inflammatory bowel diseases [11]. Nonetheless, adult stem cells have limited differentiation ability, and embryonic stem cells, despite having high differentiation potential, face ethical limitations in their acquisition process, hindering their clinical applications [58].

iPSCs, on the other hand, can be generated from various sources such as the patient’s own skin, blood, urine, etc. They possess the ability to differentiate into various cell types representing all three germ layers. Additionally, iPSCs exhibit low immunogenicity and do not involve the ethical concerns associated with embryonic stem cells, making them a novel tool for stem cell therapy research [59]. U-iPSCs have been generated from patients’ urine and differentiated into various cell types, which enables the development of patient-specific cell replacement therapies for degenerative diseases, organ failure, and tissue damage. Apart from the fundamental osteogenic, chondrogenic, and adipogenic capacity, U-iPSCs can be differentiated into alveolar type II epithelial cells [60], cardiomyocytes [61–63], fibroblasts and skeletal muscle myocytes [64], epithelial cells [35, 65], neurons and astrocytes [15, 24, 34, 65, 66], hepatocyte-like cells [67, 68], lens progenitor cells [69], retinal cell [33], and kidney precursor cells [70]. There have been studies on the utilization of u-iPSCs and their differentiated cells in the research of cell therapies of renal and neurological diseases.

Kidney disease encompasses two main types: chronic and acute. Acute kidney disease is characterized by a rapid decline in kidney function over a short period, often caused by severe infections, ischemia, drug toxicity, and other factors. Patients may experience symptoms such as oliguria, nausea, and vomiting. Chronic kidney disease, on the other hand, develops over the long term due to conditions like prolonged high blood pressure, diabetes, etc. It is a progressive condition, and patients may exhibit symptoms like fatigue, decreased appetite, and edema [71]. For those in end-stage renal disease, kidney transplantation is an effective treatment; however, challenges such as donor shortage, immune rejection, surgical complications, etc., exist [72]. Therefore, stem cell replacement therapy has emerged as a promising new approach.

Diabetic nephropathy is a form of chronic kidney disease caused by diabetes, which may eventually lead to kidney failure. Gao et al. generated u-iPSCs from urine sample of patients with diabetic nephropathy and directed their differentiation into induced nephron progenitor cells (iNPCs), which were subsequently injected into cortex of the diabetic mice’s kidney. The findings suggested that these u-iPSC derived iNPCs presented significant efficacy with reduced inflammation and fibrosis, promoted kidney regeneration and improved renal function [73]. Concerning acute kidney injury (AKI), Jin et al. established u-iPSCs from AKI patients and directed the differentiation into kidney precursor cells (KPCs). After transplantation into an ischemia–reperfusion-induced AKI mice model, the renal function was significantly ameliorated, reflected by the improvement of reduced serum creatinine and BUN levels [70].

Stress urinary incontinence (SUI) is common in women and the elderly, referring to the leakage of urine caused by an increase in abdominal pressure during activities such as coughing, sneezing, or engaging in sports. The main reason patients cannot control urine on their own is the loss of tension or dysfunction of pelvic floor muscles and the urethral sphincter due to factors such as childbirth, age, and obesity [74]. Urinary incontinence may affect the normal work and social life of patients, and the costs associated with rehabilitation and nursing services also impose a certain economic burden. Stem cell therapy may promote the repair and regeneration of damaged tissues by directing differentiation, anti-inflammatory effects, and secretion of neuroprotective factors. This approach could help improve the function of the urethral sphincter, thereby enhancing the patient’s ability to control urine. Kibschull et al. established u-iPSCs from urine of female SUI patients and differentiated them into fibroblasts and myocytes. At the three-week time point after periurethral injection into rats, these differentiated cells were traceable and found active in the periurethral areas, showing their feasibility in urethral repair and regeneration [64].

Spinal cord injury (SCI) refers to the structural and functional damage to the spinal cord caused by trauma or disease, often resulting in sensory, motor, and autonomic nervous system impairments in the areas it innervates. Severe cases may lead to disability [75]. Currently, apart from symptomatic treatment and rehabilitation measures, there is no cure for spinal cord injuries. In recent years, researchers have been exploring the role of stem cell therapy in promoting the repair and regeneration of the spinal cord [76]. Liu et al. generated neural progenitor cells (NPCs) with human u-iPSCs and transplanted NPCs into the neural tissues adjacent to lesion site of SCI rat model. The accumulation of u-iPSCs derived NPCs were observed at the lesion cavity and some differentiated into neurons, astrocytes, or oligodendrocytes, confirming the potential of u-iPSCs in nerve repair and regeneration [66].

In addition to iPSCs derived from urine, iPSCs from other cell sources have also shown potential in cell therapy for various diseases, especially neurodegenerative disorders, as demonstrated in a series of animal experiments and clinical trials [77–79]. However, before their widespread clinical application, there are still some issues related to the inherent characteristics of iPSCs that need to be addressed.

### Drug testing

#### Drug screening

U-iPSCs can be used to generate patient-specific disease models, which can then be used to screen drugs for efficacy and toxicity in a personalized manner. This can help to identify the most effective and safest treatments for individual patients. There are primarily two methods for drug screening: target-based drug screening and phenotype-based drug screening. Target-based screening is predominantly employed when there is a comprehensive understanding of the disease mechanism, and specific key enzymes, proteins, or receptors have been pinpointed. In this approach, drugs are administered with precision to evaluate their impacts on these biological molecular targets. This screening method enhances our comprehension of the precise mechanisms of drugs and aids researchers in fine-tuning drug candidates [80]. Nevertheless, as the targets often originate from idealized laboratory research models, their applicability to the intricate human body environment may be limited.

In contrast to the traditional target-based approach, phenotype-based drug screening is principally appropriate for diseases with insufficiently understood mechanisms. By observing alterations in cellular phenotypes or functions after drug treatment, the objective is to identify a drug that accomplishes the desired effects for subsequent validation and refinement. Although there are certain challenges associated with investigating specific molecular mechanisms, this method is of great value for diseases where the underlying mechanisms remain incompletely elucidated [81]. However, drugs identified through phenotype-based screening from cellular or animal models align more closely with the underlying pathological and physiological nature of the disease. This not only enhances efficiency but also increases the feasibility of drug discovery [82]. Since the introduction of iPSCs, there have been significant advancements in phenotype-based screening. Researchers achieve this by reprogramming a patient’s somatic cells into iPSCs, utilizing gene editing techniques to correct disease-associated loci within the cellular genes, thereby creating isogenic control models. After guiding iPSCs to differentiate into disease-specific cell types, drug treatments are administered, and subsequent observations of phenotypic changes in disease and control models are made to identify effective therapeutic agents [83].

u-iPSCs can be utilized to create cell lines that replicate disease phenotypes specific to individual patients, ensuring a reliable source of cell types that were previously difficult to access and expand, including neurons and cardiomyocytes. They are well-suited for conducting high-throughput drug screening to evaluate the efficacy of a wide range of pharmaceuticals. For instance, there have been studies using neural precursor cells derived from iPSCs of patients with Fragile X syndrome (FXS) to identify effective compounds which increase the expression of deficient proteins, thus providing positive proofs for FXS drug development [84–86]. Niemietz et al. generated u-iPSCs from familial amyloid polyneuropathy (FAP) patients and directed in vitro differentiation into hepatocytes. The knockdown of FAP related mutation gene transthyretin (TTR) with therapeutic oligonucleotides in u-iPSC derived hepatocytes presents high efficiency, confirming u-iPSCs as a useful tool for novel compounds screening of FAP [12]. iPSCs contribute significantly to in-depth comprehension of drug mechanisms and the identification of relevant drug targets. Furthermore, they help reduce ethical concerns related to animal experimentation while improving the efficiency of research. In general, iPSCs offer numerous advantages for phenotype-based screening. Further research is needed for the application of u-iPSCs in this area.

#### Toxicity screening

In the drug development process, comprehensive and thorough testing of drug reactivity, activity, and toxicity is crucial to ensure the effectiveness and safety of drugs once they enter the market. In the United States, approximately 60% of hospitalized patients with acute kidney injury are related to drug-induced kidney toxicity [87]. The annual socioeconomic burden resulting from drug-induced kidney toxicity can be as high as 900 million dollars [88]. The mechanisms of drug-induced kidney toxicity are complex and wide-ranging, involving various target sites such as renal tubular epithelial cells, podocytes, renal interstitium, microvascular systems [89]. In the research of drug-induced kidney toxicity mechanisms, traditional in vitro 2D cell culture models cannot effectively reflect the interactions between cells and the extracellular matrix in the in vivo microenvironment. Additionally, as cells undergo passaging, their phenotype and function may change, affecting the effectiveness and accuracy of toxicity testing. Animal models are costly, time-consuming, and raise ethical concerns. Furthermore, the differences in disease-related protein and enzyme expression between animals and humans can impact the clinical utility of drugs.

In vitro 3D culture models, such as organoids and engineered kidney tissues, consist of a diverse array of renal cell types and feature three-dimensional spatial arrangements that closely mimic the real physiological environment. Therefore, they are better suited for drug toxicity testing [90]. Our team co-cultivated USCs with Kidney-specific ECM to construct USC organoids resembling renal tubules and kidney-like organoids. Upon examination, these USC organoids exhibited a compact 3D structure with minimal central necrosis and high cell viability. They expressed specific markers such as Aquaporin-1 (AQP1) for proximal tubules, Podocin and Synaptopodin for renal glomeruli, and the secretion of erythropoietin (EPO) by renal interstitial cells. The results of drug toxicity testing showed that USC organoids were responsive to nephrotoxic drugs such as aspirin, penicillin G, acetone, and cisplatin, resulting in cell necrosis [91, 92]. Therefore, in vitro USC organoids constructed in this manner can simulate the phenotype and function of the kidney, making them suitable for studying the actual effects of drugs in a physiological environment. Kidney-like organs derived from iPSC sources contain a greater variety of cell types at different developmental stages. Further development of organoids based on u-iPSCs may result in models that are more suitable for toxicity testing [93].

**Fig. 3:**
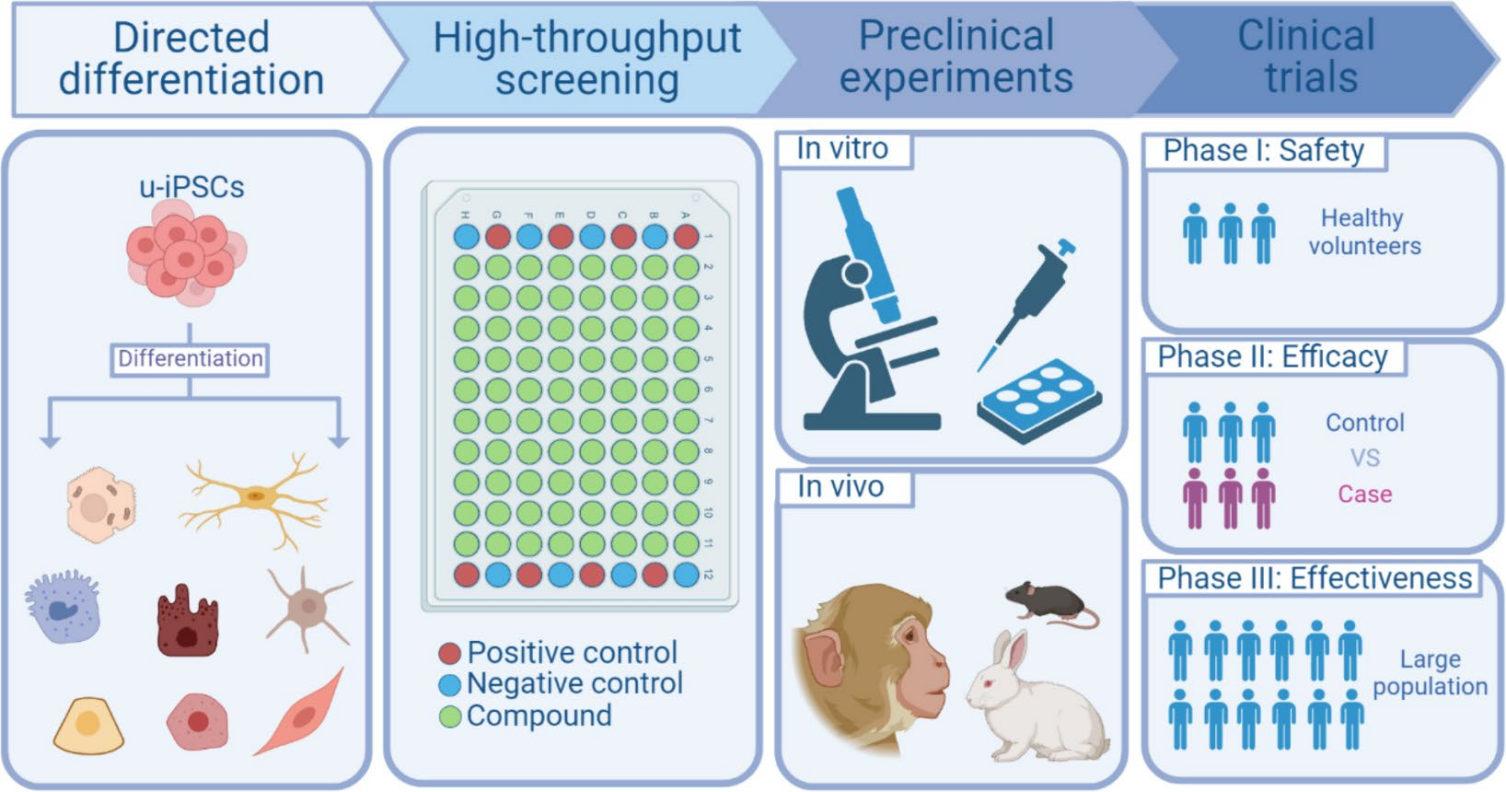
The process of drug discovery with patient-specific u-iPSCs. After the generation from patients’ urine, U-iPSCs are differentiated into specific cell types relevant to the disease. Subsequently, the high-throughput screening is conducted, where thousands of chemical compounds or drugs are rapidly tested to identify potential candidates that have a desired effect on the cells. These selected compounds are then subjected to preclinical experiments and clinical trials to assess their efficacy and safety. (Created with BioRender.com)

Mitochondrial dysfunction plays a significant role in the mechanisms of drug toxicity. Drugs induce damage and functional impairment of mitochondria through various mechanisms, including inhibiting mitochondrial replication, affecting the electron transport chain responsible for ATP synthesis, altering mitochondrial permeability, and inhibiting the function of mitochondrial membrane transport proteins. In highly metabolic organs like the heart, kidney, skeletal muscle, and in the liver when drug concentrations are high, drug-induced mitochondrial damage not only leads to organ toxicity but can also result in symptoms such as increased glycolysis and lactic acid accumulation, leading to acidosis [94]. Therefore, the assessment of in vivo and in vitro mitochondrial toxicity is an essential component of drug safety evaluation in the drug development process. We developed 3D USC spheroids to assess the chronic cytotoxicity and mitochondrial toxicity of anti-retroviral drugs, including zalcitabine, tenofovir, and Raltegravir. The results showed that these drugs inhibited the expression of certain mitochondrial oxidative phosphorylation enzymes and reduced mitochondrial DNA content [95]. Furthermore, we seeded USCs onto silk fibers to construct three-dimensional tissue-engineered structures and treated them with anti-retroviral drugs. The results demonstrated that this model could more sensitively reflect the effects of drugs on mitochondria compared to 3D USC spheroids [96] (Fig. 3).

### Disease modeling

For some rare and genetic diseases, the scarcity of clinical samples and the difficulty in establishing animal models have posed challenges to the study of their specific molecular mechanisms. USCs, as an excellent source of cells that can be non-invasively and conveniently obtained in large quantities from patients, can be reprogrammed into u-iPSCs and further differentiated into the cell types relevant for studying the diseases [29]. Many research teams have successfully utilized patient-derived u-iPSCs in conjunction with gene editing technologies to construct cell models for various human systems’ diseases. This serves as a powerful tool to help researchers better understand the disease mechanisms and develop new therapeutic strategies (Table 1, see the end of the main text).

**Table 1:** Cellular diseases models of various systems established with u-iPSCs

Disease	Inheritance pattern (responsible gene)	Reprogramming techniques	Generated cell type	Phenotype
Duchenne muscular dystrophy (DMD) [62, 97]	X-linked recessive disorder (DMD gene)	Episomal vectors	iPSCs; Induced cardiomyocytes	iPSCs: Round colonies of tightly packed cells with large nuclei; Positive pluripotency markers: OCT-4, TRA-1-60, SSEA-4; Negative marker: SSEA-1. iPSC-derived Cardiomyocytes: Positive cardiac markers NKX2-5 and TNNT-2
X-linked Alport syndrome (X-LAS) [98]	X-linked dominant inheritance (COL4A5 gene)	Sendai virus	iPSCs	Positive pluripotent markers: OCT4, SOX2, NANOG, LIN28; Normal karyotype; Differentiation into three germ layers
Cryptorchidism [99]	- (INSL3, ZNF 214, and ZNF215)	Lentiviruses	iPSCs	Morphology, marker expression, and epigenetic status of OCT4 similarity to embryonic stem cells; Differentiation into three germ layers; Normal karyotype; Positive pluripotent markers: OCT4, SOX2, NANOG, LIN28
Ventricular septal defects (VSDs) [61]	- (RyR2 gene)	Sendai virus	iPSCs; Induced cardiomyocytes	iPSCs: Alkaline phosphatase activity; RyR2 mutation retention; Pluripotency marker expression; Differentiation into three germ layers. iPSC-derived cardiomyocyte: Hallmarks; Spontaneous contraction; Expression of cardiac-specific markers: GATA4, IRX4, cTnT, cTnI, ACTN2, MLC-2a, and MLC-2v
Hemophilia A/B [67, 100–102]	X-linked recessive disorder (FVIII/FIV gene)	Episomal vectors; Retroviral vectors	iPSCs; Induced hepatocyte-like cells; Induced endothelial cells	Positive hepatocyte markers SOX17, HNF4, ALB, HNF4α, AFP, A1AT, ALB; Positive endothelial cell markers CD31 and CD144
Thalassemia [103]	Single-gene recessive hemoglobin disorder (Globin gene)	Episomal vectors	iPSCs	Positive pluripotent markers: OCT4, NANOG, SOX2, OCT4, and TRA-1-60; Normal karyotype; Globin gene mutation
Down syndrome (DS) [34]	- (trisomy of human chromosome 21)	Episomal vectors	iPSCs; Induced neurons and astrocytes	Neuron and astrocyte differentiation; Cortical organoid formation mimicking early cortical development; Neural progenitor zone organization; Excitatory and inhibitory neuron differentiation; Upper- and deep-layer cortical neuron generation; Astrocyte formation
Autism spectrum disorder (ASD) and developmental delay (DD) [104]	- (AUTS2 gene)	Episomal vectors	iPSCs	AUTS2 deletion; Normal karyotype; Differentiation potential; Positive pluripotency markers: OCT4, SOX2 and NANOG
X-linked Renpenning syndrome (X-RSY) [105]	X-linked disorder (PQBP1 gene)	Sendai virus	iPSCs	Normal karyotype; Differentiation potential; Positive pluripotency markers: OCT4, NANOG,
Hereditary deafness [106]	- (TMC1 gene)	Episomal vectors	iPSCs	Normal karyotype; Differentiation potential; Positive pluripotency markers: OCT4, SOX2, and NANOG
Attention Deficit Hyperactivity Disorder (ADHD) [107]	Unknown	Sendai virus	iPSCs	Normal karyotype; Differentiation potential; Positive pluripotency markers: TRA-1-60, Sox2, Oct3/4 and SSEA4
Sporadic Alzheimer’s disease (sAD) [108]	Sporadic (APOE, BIN1, MS4A6A gene)	NM-RNA transfection	iPSCs	Normal karyotype; Differentiation potential; Positive pluripotency markers: NANOG, OCT4, and SOX2
Spinocerebellar ataxia type 3 (SCA3) [109]	Autosomal dominant genetic syndrome (ATXN3 gene)	Sendai virus	iPSCs	Normal karyotype; Differentiation potential; Positive pluripotency markers: OCT4, Nanog, TRA-1-60
Neurofibromatosis type 1 (NF1) [110]	Autosomal dominant genetic syndrome (neurofibromin gene)	Episomal vectors	iPSCs	Normal karyotype; Differentiation potential; Positive pluripotency markers: OCT4, NANOG and SOX2
Brain tumors [111]	Unknown	Sendai virus	iPSCs; Induced mesenchymal stem cells (iMSCs)	iPSCs: Positive pluripotent markers; Differentiation potential; Normal karyotype. iMSCs: Positive MSC markers: CD13, CD29, CD73, CD90 and CD105; Immunomodulatory properties
Osteogenesis imperfecta (OI) [112]	Single gene inherited disorder (COL1A1 gene)	Episomal vectors	iPSCs	Normal karyotype; Differentiation potential; Positive pluripotency markers: SSEA4, OCT4, TRA-1-60 and TRA-1-81
Autosomal dominant osteopetrosis type II (ADO2) [113]	Autosomal dominant genetic disorder (CLCN7 gene)	Episomal vectors	iPSCs	CLCN7 mutation retention; Normal karyotype; Differentiation potential; Positive pluripotency markers: NANOG, TRA-1-60, OCT4, TRA-1-81, SOX2, and SSEA4
Fibrodysplasia ossificans progressiva (FOP) [114, 115]	Autosomal dominant genetic disorder (ACVR1 gene)	Sendai virus	iPSCs; Induced endothelial cells (ECs) and pericytes	iPSCs: R206H mutation retention; Normal karyotype; Differentiation potential; Positive pluripotency markers: OCT4, TRA-1-81, and SSEA4. Induced ECs: Positive markers: CD31 in ECs and PDGFRb in pericytes
X-linked adrenoleukodystrophy (X-ALD) [116]	X-linked disorder (ABCD1 gene)	Sendai virus	iPSCs	Normal karyotype; Differentiation potential; Positive pluripotency markers: OCT4, SOX2, NANOG, LIN28
Methylmalonic acidemia (MMA) [117]	Autosomal recessive disease (MMACHC gene)	Episomal vectors	iPSCs	Normal karyotype; Differentiation potential; Positive pluripotency markers: NANOG, OCT4, SOX2 and SSEA4
Phenylketonuria (PKU) [118]	Autosomal recessive disease (PAH gene)	Episomal vectors	iPSCs	Normal karyotype; Differentiation potential; Positive pluripotency markers: OCT4, SOX2, and NANOG
Barth syndrome (BTHS) [119]	X-linked autosomal recessive disease (TAZ gene)	Sendai virus	iPSCs	Normal karyotype; Differentiation potential; Positive pluripotency markers: NANOG, OCT4, SOX2, SSEA4
Autosomal dominant hypercholesterolemia (ADH) [68]	Autosomal dominant genetic disorder (PCSK9 gene)	Episomal vectors	iPSCs; Induced hepatocyte-like cells	iPSCs: PCSK9 mutation retention; Differentiation potential; Positive pluripotency markers: TRA-1-60, SSEA3, SSEA4. Induced hepatocyte-like cells: Positive hepatic markers FOXA2, AFP, HNF4α, albumin, SREBF2, and LDLR
Systemic lupus erythematosus (SLE) [120]	Complex genetic factors	Lentiviruses	iPSCs	Normal karyotype; Differentiation potential; Positive pluripotency markers: OCT3/4, SOX2, GDF3, TERT, KLF4, c-MYC, and NANOG
Ankylosing spondylitis (AS) [121]	Complex genetic factors (JAK2 gene)	Episomal vectors	iPSCs	JAK2 mutation retention; Normal karyotype; Differentiation potential; Positive pluripotency markers: NANOG, TRA-1-60, OCT4
X-linked juvenile retinoschisis (XLRS) [122]	X-linked disorder (RS1 gene)	Sendai virus	iPSCs	RS1 mutation retention; Normal karyotype; Differentiation potential; Positive pluripotency markers: NANOG, SOX2, OCT4
Retinitis pigmentosa (RP) [123]	Autosomal dominant genetic disorder (PRPF8 gene)	Episomal vectors	iPSCs	PRPF8 mutation retention; Normal karyotype; Differentiation potential; Positive pluripotency markers: OCT4, SOX2 and NANOG

*Abbreviations: OCT-4 *Octamer binding transcription factor-4,* TRA-1-60 *Tumour resistance antigen 1–60, *SSEA-4 *Stage specific embryonic 4 antigen,* NKX2-5 *NK2 home box 5,* TNNT-2 *Gene for cardiac Troponin T,* ABCD1 *ATP-binding-cassette transporter subfamily D member 1,* INSL3 *Insulin-like factor 3,* ZNF *Zinc finger,* RyR2 *Ryanodine receptor 2,* GATA4 *GATA Binding Protein 4,* IRX4 *Iroquois Homeobox 4,* cTnT *Cardiac troponin T,* cTnI *Cardiac troponin I,* ACTN2 *a-actinin,* MLC-2a *Myosin light chain,* MLC-2v *Myosin light chain-2,* FOXG1 *Forkhead box G1,* SYN-1 *synapsin-1,* MAP2 *Microtubule-associated protein 2,* NMDAR *N-methyl-D-aspartate receptor,* GS *Glutamine synthetase,* CD44 *Cluster of differentiation 44,* GFAP *Glial fibrillary acidic protein,* C4 *Complement system component 4,* VGLUT1 *Vesicular glutamate transporter-1,* GABAAR *Gamma-aminobutyric acid type A receptor,* PQBP1 *Polyglutamine binding protein 1 gene,* ACVR1 *Activin receptor-like kinase 2,* VEGFR2 *Vascular endothelial growth factor receptor 2,* PAH *Phenylalanine hydroxylase, *TAZ *tafazzin, *PCSK9 *Proprotein convertase subtilisin kexin type 9, *TERT *Telomerase, *JAK2 *Janus kinase 2, *RS1 *Retinoschisin, *PRPF8 *Pre-mRNA processing factor 8

#### Muscular disease

Duchenne muscular dystrophy (DMD) is a hereditary muscle disease caused by mutations in the DMD gene on the X chromosome. DMD patients typically experience a lack or mutation in the DMD gene, preventing the normal production of the encoded muscle protein. This leads to damage in muscles such as skeletal, cardiac, and respisiratory muscles. Many DMD patients gradually develop movement disorders due to progressive muscle degeneration, and in severe cases, it can impact the heart and respiratory system, ultimately resulting in the patient’s death. Research indicates that, on average, four out of every five DMD patients succumbs to heart failure or respiratory failure [124]. To investigate this disease, Ghori et al. extracted urinary stem cells (USCs) from Pakistani children and efficiently reprogrammed them into u-iPSCs through transfection with episomal vectors [97]. Subsequently, after 11 days of in vitro induction, u-iPSCs successfully differentiated into DMD-Cardiomyocytes expressing cardiac markers such as NKX2-5 and TNNT-2 [62]. The establishment of this DMD cell model lays the foundation for further research into the molecular mechanisms of DMD and the identification of drug targets.

Ventricular Septal Defects (VSDs) is a common congenital heart disease. An abnormal opening in the ventricular septum allows the mixing of oxygenated and deoxygenated blood, causing the heart to pump blood more strenuously. This can ultimately lead to various complications, including symptoms such as shortness of breath, fatigue, and heart failure (HF) [125].

Cao et al. generated u-iPSCs with the ryanodine receptor type 2 (RyR2) mutation from a 2-month-old male patient with VSD with HF and directed the differentiation into functional cardiomyocytes by temporally manipulating canonical Wnt signaling using small molecules [61]. This study provides a robust cell model for investigation of the pathogenesis of VSD with HF.

#### Genitourinary disease

As cells derived from the kidney, USCs have been utilized in constructing models for kidney diseases [93, 126, 127]. X-linked Alport Syndrome (X-LAS), primarily caused by mutations in the gene encoding the protein COL4A5 in the renal tubular basement membrane, is an inheritable disorder affecting the renal tubular basement membrane. Damage to the renal tubular basement membrane leads to glomerulosclerosis and renal failure, resulting in clinical manifestations such as proteinuria, hematuria, and hypertension. Additionally, complications may involve the eyes and inner ears [128, 129]. Guo et al. established a u-iPSC line with USCs harvested from a 5-year-old male X-LAS patient and demonstrated the feasibility as a cell-based disease models by verifying the expression of pluripotent makers, normal karyotype and capacity to differentiate into multiple germ layers [98].

A series of pediatric diseases are genetic in nature, including cystic fibrosis, congenital heart disease, spinal muscular atrophy, and others [130–132]. In the study of pediatric disease mechanisms, obstacles in obtaining early human embryos and associated ethical concerns have been significant limiting factors, highlighting the need for an appropriate in vitro research model. Cryptorchidism is a congenital reproductive system disorder characterized by the failure of the male testes to descend properly into the scrotum during embryonic development. Untreated cryptorchidism may lead to complications such as infertility and testicular cancer, posing reproductive health risks for affected individuals [133]. Zhou et al. reprogrammed USCs from a cryptorchid patient with mutations in genes including insulin-like factor 3 (INSL3), zinc finger (ZNF) 214, and ZNF215 into u-iPSCs [99]. By comparing them with human embryonic stem cells, the study confirmed their phenotypic, karyotypic, and pluripotent differentiation capabilities, providing a valuable in vitro model for understanding the disease mechanisms.

#### Blood disorder

Hemophilia is a common genetic bleeding disorder. The two most prevalent types are Hemophilia A and Hemophilia B, resulting from mutations in the F8 gene on the X chromosome and the F9 gene, respectively, leading to deficiencies in clotting factors VIII and IX [134]. Due to abnormalities in the clotting process, individuals with severe hemophilia may face life-threatening excessive bleeding during injuries or surgeries. The primary treatment involves replacing the deficient clotting factors by injecting plasma or preparations containing these factors [135]. However, one drawback is the development of antibodies, reducing the clinical effectiveness.

To advance gene therapy and novel clotting factor development, establishing appropriate disease models is crucial. Lu et al. generated u-iPSCs from a Hemophilia A patient with an Inv22 mutation through the electroporation of USCs using episomal plasmids [100]. Similarly, Ma et al. produced iPSCs from a Hemophilia B patient carrying the F9 variant c.223 C > T (p.R75X) [101]. The establishment of Hemophilia A and Hemophilia B iPSC lines serves as a robust tool for comprehending the underlying molecular mechanisms of hemophilia. In the studies by two other teams, they established Hemophilia A iPSC lines using USCs obtained from patient urine, and subsequently differentiated them in vitro into liver cells [67] and endothelial cells [102] with patient-specific mutations. Apart from hemophilia, the u-iPSC lines of another hemoglobin disorder, thalassemia, have been generated from patients carrying different mutations on globin genes [103]. The u-iPSC lines of various blood disorders provide a valuable cellular source for gene-corrected cell therapy.

#### Neurological disease

In the research of neurological disorders, the establishment of existing cell and animal disease models has provided powerful tools for studying the pathogenic mechanisms. However, the etiology of neurodevelopmental and neurodegenerative diseases is diverse, involving complex interactions between genetic and environmental factors that cannot be fully simulated in animal models. Some peripheral neuromuscular diseases may require sampling from patients, but obtaining samples of brain and spinal cord tissue is clinically challenging [136]. Therefore, finding suitable stem cells to establish disease models for in vitro dynamic and continuous research is crucial for understanding the occurrence and development of neurological disorders.

Neurodevelopmental disorders (NDDs) refer to defects or abnormalities in the early development of the central nervous system, leading to impaired functions such as behavior and cognition in patients. The manifestations of neurodevelopmental disorders often appear in preschool children, and their impact can last a lifetime, with most cases lacking clear treatment options [137]. U-iPSC lines have been established using urine from patients with NDDs, including autism spectrum disorder (ASD), developmental delay (DD), X-linked Renpenning syndrome (X-RSY), Down Syndrome, Attention Deficit Hyperactivity Disorder (ADHD), and TMC1-related hereditary deafness [34, 104–107]. Teles et al. created three-dimensional human cerebral organoids with neurons and astrocytes differentiated from u-iPSCs derived from Down syndrome (DS) patients [34], which demonstrated developmental dynamics of the early-stage forebrain.

Neurodegenerative disorders (NDDs) involve the gradual degeneration of neurons in the brain and spinal cord, leading to irreversible cognitive impairments, motor dysfunction, and other symptoms [138]. Patient-specific u-iPSC lines for several neurodegenerative disorders, such as Alzheimer’s disease (AD) and Spinocerebellar ataxia type 3 (SCA3), have been developed in recent years. Sporadic Alzheimer’s disease (sAD), the most common form of dementia, predominantly influenced by genetic factors such as single nucleotide polymorphisms (SNPs). An iPSC line, KEIOi005-A, derived from USCs of a mild Alzheimer’s disease patient carrying multiple sporadic Alzheimer’s disease risk SNPs, exhibits normal stemness and pluripotency, and was suitable for in vitro modeling of sAD [108]. Spinocerebellar ataxia type 3 (SCA3), a neurodegenerative condition caused by a CAG repeat expansion in the ATXN3 gene, leads to progressive ataxia affecting balance, gait, and speech. The transformation of USCs from SCA3 patients into iPSCs hints at the potential of the ZZUi004-A iPSC line for studying SCA3’s underlying mechanisms, facilitating drug trials, and investigating gene therapy approaches [109].

In addition to NDDs, the establishment of u-iPSC lines for a movement disorder, paroxysmal kinesigenic dyskinesia (PKD), a tumor predisposition syndrome, neurofibromatosis type 1 (NF1), and brain tumor also demonstrates the value of u-iPSCs in modeling various neurological diseases [110, 111, 139]. Paroxysmal Kinesigenic Dyskinesia (PKD) is a genetic movement disorder linked to mutations in the PRRT2 gene. Disease-specific iPSCs generated from USCs from a PKD patient with a specific mutation present reduced PRRT2 expression and can differentiate into neurons. However, electrophysiological examinations find no significant differences compared to control cells. Overall, the study suggests that u-iPSCs offer a valuable tool for investigating PKD’s mechanisms [139]. Another study investigates using urine samples to generate iPSCs from pediatric brain tumor patients. These brain tumor iPSCs closely resemble iPSCs from non-tumor patients in terms of characteristics and ability to differentiate. Both types of iPSCs can efficiently turn into functional induced mesenchymal stem/stromal cells (iMSCs) with immunomodulatory properties, suggesting a promising non-invasive approach for personalized iMSC-based treatments for pediatric brain tumors [111].

#### Skeletal disorder

Musculoskeletal diseases rank second among global disabling conditions, imposing a significant burden on society [140]. As one of the most prevalent genetic diseases, hereditary musculoskeletal disorders can lead to fractures, muscle injuries, limited joint mobility, restricting patients’ daily activities, and diminishing their quality of life. In the exploration of the genetic factors underlying musculoskeletal disorders, researchers face challenges such as difficulties in obtaining samples from the human body and the lack of well-established models for rare diseases. The establishment of patient-derived iPSC lines and the in vitro directed osteogenesis provide suitable models for studying musculoskeletal diseases.

Several research teams have utilized u-iPSCs in the disease modeling of various genetic bone disorders, including Osteogenesis Imperfecta (OI) [112], Autosomal Dominant Osteopetrosis Type II (ADO2) [113], and Fibrodysplasia Ossificans Progressiva (FOP) [114, 115]. OI is caused by mutations in collagen genes, resulting in bones that are prone to fractures, often accompanied by other connective tissue issues. Luan et al. generated an iPSC line from USCs of a 15-year-old female OI patient with a COL1A1 gene mutation using integration-free episomal vectors [112]. ADO2, a dominant inherited musculoskeletal disorder, leads to fractures, joint pain, and changes in bone morphology. Ou et al. produced ADO2-iPSCs from USCs of an ADO2 patient and identified the same CLCN7 mutation (R286W) present in the patient’s blood samples by comparing them with ADO2-iPSCs [113]. FOP is a rare disease caused by mutations in the ACVR1 gene, resulting in the gradual ossification of soft tissues, leading to loss of joint function and restricted movement. Two research groups generated iPSC lines with USCs from FOP patients carrying R206H mutations [114, 115]. Cai et al. further directed the differentiation of FOP-iPSCs into endothelial cells and pericytes, revealing disease-related phenotypes in vitro [114].

#### Metabolic disorder

Inherited Metabolic Disorders (IMD) are caused by genetic mutations that result in structural and functional changes in the encoded protein molecules [141]. This leads to abnormalities in biochemical reactions and metabolism, with the accumulation of intermediate metabolites in the body, causing a range of clinical manifestations. IMDs have varied onset times, can affect multiple organs, and exhibit diverse clinical presentations [142]. Most IMDs currently lack effective treatments.

U-iPSCs have been used to establish disease models for various IMDs. Peroxisomes aid in the breakdown of fatty acids and hydrogen peroxide in the human body. When deficient, the accumulation of fatty acids and hydrogen peroxide can damage various tissues and organs. X-linked Adrenoleukodystrophy (X-ALD) is a hereditary metabolic disorder caused by peroxisomal dysfunction. It leads to progressive neurodegeneration, movement disorders, cognitive impairment, vision loss, adrenal insufficiency, and other symptoms. Wang et al. generated a u-iPSC line from a 6-year-old X-ALD patient with an ABCD1 gene mutation [116]. Additionally, IMDs related to amino acid metabolism, such as methylmalonic acidemia (MMA) and phenylketonuria (PKU), result from the deficiency of enzymes involved in their metabolism, leading to the accumulation of intermediate products. Han’s research group generated u-iPSC lines from a 10-year-old male MMA patient [117] and a 15-year-old male PKU patient [118]. Other u-iPSC lines for IMDs, including Barth syndrome and autosomal dominant hypercholesterolemia (ADH), have also been generated, providing a powerful tool for further understanding metabolic diseases [68, 119].

#### Autoimmune disease

In autoimmune diseases, the immune system attacks the body’s own normal tissues, causing inflammation and damage to multiple organs throughout the body [143]. Autoimmune diseases often have multifaceted causes, including genetic, environmental, and immune system factors, making the design and implementation of clinical trials challenging. Establishing ideal animal or in vitro models and precisely regulating the immune system to minimize side effects are also challenges in research. Systemic Lupus Erythematosus (SLE) and Ankylosing Spondylitis (AS) are two distinct rheumatic diseases. The former can involve various organs throughout the body, causing symptoms such as rash, fatigue, and fever [144]. The latter primarily affects the spine and pelvic joints, manifesting as lower back pain and morning stiffness [145]. Chen et al. and Hu et al. generated u-iPSC lines from SLE patients and an AS patient with a JAK2 mutation, respectively, confirming USCs as an ideal source for modeling autoimmune diseases [120, 121].

#### Retinal disorder

Inherited retinal diseases (IRDs) are a group of diseases characterized by progressive changes in the retina leading to vision loss, including X-linked Juvenile Retinoschisis (XLRS), Retinitis Pigmentosa (RP), and others. The global prevalence of monogenic IRDs is approximately 1 in 2,000, making them a significant cause of irreversible blindness in children and the working-age population [146]. Gene therapy is currently the only effective treatment for such diseases, and there is an urgent need for more experimental research and clinical trials to provide new therapeutic approaches. U-iPSCs have been used in modeling some inherited retinal diseases. Tang’s research group, for example, established two u-iPSC lines from individuals with specific conditions: an 11-year-old male with XLRS carrying a mutation in the retinoschisin gene (RS1) and a 17-year-old male patient with RP harboring a mutation in the pre-mRNA processing factor 8 gene (PRPF8) [122, 123].

## Epigenetic memory in u-iPSCs’ differentiation

Reports indicate that iPSCs derived from various somatic sources exhibit distinct epigenetic signatures, which influence their differentiation potential towards specific cell lineages associated with the donor tissue while hindering others. This “epigenetic memory” from the donor tissue may impede iPSC reprogramming efficiency and their ability to differentiate into desired cell types for disease modeling and treatment [147]. The impact of epigenetic memory on the differentiation of u-iPSCs is complex. Despite expressing mesenchymal stem cell (MSC) markers, u-iPSCs exhibit properties similar to parietal epithelial cells. While u-iPSCs can effectively differentiate into various cell lineages, they show a stronger propensity towards renal and epithelial cell types with tight junction and barrier function, indicating a nuanced view of epigenetic memory in u-iPSCs [148, 149]. Although there are no observed negative effects on differentiation, further investigation into the underlying mechanisms and long-term consequences is needed.

Direct reprogramming reduces the risk of teratogenesis due to the lack of a pluripotent intermediate state and holds the potential of preserving the epigenetic memory of the donor cell, which has a tremendous impact on the accuracy of disease modeling [150]. By reprogramming USCs into iPSCs and subsequently directing the differentiation, the full course could extend to more than 12 weeks. Comparatively, through direct reprogramming, following expansion for 3 to 4 weeks, USCs undergo transduction with inducible MyoD (iMyoD) lentivirus, differentiate, and form myotubes in approximately 8 weeks. The shortening of culture time leads to reduced cost losses and increased efficiency, which offers an efficient and cost-effective method for generating patient-specific cell lineages [147].

Despite the advantages, optimization is required to enhance the efficiency of directed differentiation of USCs for generating target cells. Also, mature cells differentiated from reprogrammed USCs need thorough evaluation through genetic, biological, and functional assessments [151]. USCs exhibit potential for superior differentiation, making them valuable for studying mechanisms underlying both common and rare genetic diseases, as well as for drug screening purposes [152]. Future research should focus on understanding the specific epigenetic marks associated with different cell lineages, improving reprogramming techniques, optimizing lineage-specific differentiation protocols, and identifying pathways, growth factors, and culture conditions to overcome potential biases and enhance therapeutic applicability.

## Challenges and considerations

Although u-iPSCs hold immense promise for precision medicine, several challenges and considerations must be addressed to fully exploit their potential. iPSCs exhibit high pluripotency, but their sensitivity to reprogramming varies depending on different cell sources, and their growth curves and differentiation tendencies may differ. Additionally, variations in reprogramming factor concentrations, types of transfection methods, cell culture conditions, and timing among different laboratories may lead to reduced iPSC induction efficiency and even the generation of off-target cells [153]. Therefore, establishing standardized reprogramming protocols is crucial to ensure the reproducibility and comparability of experiments across different research teams, which is key to improving the accuracy of experimental results. Standardization measures may include using the same cell source, selecting a set of standard classical reprogramming factors, maintaining consistency in experimental conditions, and establishing uniform iPSC identification criteria.

iPSCs have the ability for unlimited proliferation, but different cell lines have different mutation rates. Some genetic mutations may be introduced during iPSC reprogramming and amplification, leading to the occurrence of tumors [154]. Therefore, maintaining genomic stability of iPSCs during long-term expansion is crucial for ensuring their safety. Researchers conduct differentiation status checks, sequencing, and karyotype analysis on generated iPSCs to eliminate possible variations. Other methods include using non-integrative reprogramming methods, employing gene editing techniques to repair potential tumorigenic mutations in iPSCs, and pre-differentiating iPSCs into specific cell types in vitro [155]. In summary, stringent quality control and safety measures must be applied to iPSCs before clinical application to eliminate their tumorigenic potential.

In many current experimental results, iPSCs do express classical markers and possess specific morphological features. However, they may not function well in vivo. On one hand, iPSCs may aberrantly differentiate into teratomas, causing immune rejection. Moreover, the survival and engraftment of iPSCs in vivo require suitable conditions, and simple cell injections may not provide the appropriate microenvironment to promote their in vivo differentiation and maturation [156]. Therefore, researchers can consider a series of measures, including choosing the right treatment timing, an adequate number of cells, using biomaterials as scaffolds for iPSCs, and pre-differentiating them in vitro.

After addressing a series of laboratory issues, the goal is to achieve large-scale production of iPSCs to meet clinical needs. First, the selection of an appropriate cell source, such as USCs, which can be easily obtained in large quantities non-invasively, is crucial for large-scale expansion. Then, non-integrative reprogramming methods or gene editing techniques need to be adopted, along with optimized cell expansion strategies, and the establishment of scalable, automated culture systems to improve cell production efficiency. Additionally, regular testing and screening of iPSCs, timely removal of abnormal cells, and ensuring cell quality are essential. Finally, efficient purification and obtaining the desired cell types, along with the establishment of cell cryopreservation and recovery processes, are necessary for iPSCs to be used promptly when needed. Considering these steps collectively, pharmaceutical companies can mass-produce iPSCs, providing better tools for drug screening, disease modeling, and cell therapy [157].

## Conclusion

U-iPSCs are a powerful tool for advancing precision medicine. Their unique advantages, such as non-invasive acquisition and high reprogramming efficiency, make them a promising source of patient-specific pluripotent cells for drug testing, disease modeling, and cell therapy. With further research and development, u-iPSCs have the potential to revolutionize the treatment of a wide range of diseases and improve healthcare outcomes for millions of patients. Overall, the review manuscript provides a comprehensive and insightful overview of the potential and application of u-iPSCs in precision medicine. It is evident that u-iPSCs are a powerful tool for advancing personalized healthcare and improving patient outcomes.

## Data Availability

Not applicable.
